# Regulatory Mechanisms of the RNA Modification m^6^A and Significance in Brain Function in Health and Disease

**DOI:** 10.3389/fncel.2021.671932

**Published:** 2021-05-19

**Authors:** Justine Mathoux, David C. Henshall, Gary P. Brennan

**Affiliations:** ^1^Department of Physiology and Medical Physics, RCSI, University of Medicine and Health Sciences, Dublin, Ireland; ^2^FutureNeuro SFI Research Centre, RCSI, University of Medicine and Health Sciences, Dublin, Ireland; ^3^UCD School of Biomolecular and Biomedical Science, UCD Conway Institute, University College Dublin, Dublin, Ireland

**Keywords:** m6A (N6-methyladenosine), brain function and brain diseases, brain development, epitranscriptomics, METTL3

## Abstract

RNA modifications have emerged as an additional layer of regulatory complexity governing the function of almost all species of RNA. *N*^6^-methyladenosine (m^6^A), the addition of methyl groups to adenine residues, is the most abundant and well understood RNA modification. The current review discusses the regulatory mechanisms governing m^6^A, how this influences neuronal development and function and how aberrant m^6^A signaling may contribute to neurological disease. M^6^A is known to regulate the stability of mRNA, the processing of microRNAs and function/processing of tRNAs among other roles. The development of antibodies against m^6^A has facilitated the application of next generation sequencing to profile methylated RNAs in both health and disease contexts, revealing the extent of this transcriptomic modification. The mechanisms by which m^6^A is deposited, processed, and potentially removed are increasingly understood. Writer enzymes include METTL3 and METTL14 while YTHDC1 and YTHDF1 are key reader proteins, which recognize and bind the m^6^A mark. Finally, FTO and ALKBH5 have been identified as potential erasers of m^6^A, although there *in vivo* activity and the dynamic nature of this modification requires further study. M^6^A is enriched in the brain and has emerged as a key regulator of neuronal activity and function in processes including neurodevelopment, learning and memory, synaptic plasticity, and the stress response. Changes to m^6^A have recently been linked with Schizophrenia and Alzheimer disease. Elucidating the functional consequences of m^6^A changes in these and other brain diseases may lead to novel insight into disease pathomechanisms, molecular biomarkers and novel therapeutic targets.

## Introduction

The brain is the most complex and cellularly diverse organ in the body. Higher order brain functions, and those functions critical to sustain life are facilitated by the concerted activity of different cell types, each functionally driven by distinct, context dependent gene expression and gene expression regulation patterns (Hawrylycz et al., 2012; Mitra et al., 2021). Recently, the application of single cell RNA-sequencing technologies has begun to reveal at single cell resolution this dynamic and distinct gene expression patterns and how they respond to stimulus or activity (Agarwal et al., 2020; Pfisterer et al., 2020; Joglekar et al., 2021; Song et al., 2021).

Appropriate spatiotemporal gene expression in the brain involves several tightly monitored layers of regulation. Broad effector transcription factors direct transcription of genes via canonical binding sites throughout the genome, a process known to be critical for learning and memory and memory consolidation (Kaldun and Sprecher, 2019). Epigenetic mechanisms (DNA methylation, histone modifications etc.), often in response to transcription factor signals, modify DNA and associated histone structure to enhance or repress transcription (Kim et al., 2009; Conboy et al., 2021). Post-transcriptional mechanisms, including microRNAs, are also known to profoundly influence gene expression (or rather mRNA translation) in the brain adding an additional layer of regulatory complexity and fine-tuning of gene output (Bartel, 2018; Brennan and Henshall, 2020). The proteome is also tightly regulated and post-translational modifications such as phosphorylation, ubiquitylation and sumoylation dictate the efficacy or function of the resulting protein according to the needs of a given cell (Zhang et al., 2016; Czuba et al., 2018). These processes are not independent of each other and function cohesively, cumulatively forming a complex control over gene output.

RNA is now widely recognized to undergo complex post-transcriptional editing and modification (in addition to alternative splicing and polyadenylation) which confer additional information carrying capacity and profoundly influence its fate (Jain et al., 2019; Zaccara et al., 2019; He and He, 2021). Among the most well studied involves adenosine to inosine (A-to-I) conversion which is mediated by both ADAR I and II enzymes (Eisenberg and Levanon, 2018; Christofi and Zaravinos, 2019). A-I editing involves the hydrolytic deamination of adenosine to inosine. This recoding can result in non-synonymous amino acid substitutions, resulting in altered protein-coding sequences and potentially altered protein structure (Eisenberg and Levanon, 2018). Additionally, recoding of the RNA sequence within the 3′-UTR may alter mRNA-translational efficiency as it may affect microRNA-mediated targeting (Brummer et al., 2017; van der Kwast et al., 2020). Other modifications are more subtle than overt structural modification of bases, involving covalent modification of RNA such as the addition of methyl groups to specific nucleotides. Various forms of RNA were known to undergo extensive methylation as far back as the 1970s (Munns et al., 1977; Wei and Moss, 1977; Burke and Joh, 1982). However, recent advances in transcriptomic technologies and the development of antibodies, which specifically target these modifications have made it possible to reveal the extent and identity of these novel RNA modifications (Dominissini et al., 2012; Meyer et al., 2012; Dominissini et al., 2013). Critically, many of these modifications may be dynamic in nature undergoing context-dependent addition and removal.

The addition of methyl groups to the N6 position of adenosine [N6-methyladenosine (m^6^A)] is the most abundant internal modification in RNA and is prevalent in brain tissue. However, the function of m^6^A in brain is only beginning to emerge. In this review, we provide an overview of the mechanisms by which m^6^A is regulated and try to define its overall contribution to the gene expression landscape in brain cells. We also discuss recent reports, which detail the involvement of m^6^A in neurological diseases.

## M^6^A Rna Methylation

It was identified in the 1970s that certain forms of RNA such as rRNA undergo extensive methylation (Munns et al., 1977; Wei and Moss, 1977). However, it was thought to be mainly structural and further interrogation was limited by the technology at this time (Furuichi et al., 1975). The development of an antibody which recognizes the m^6^A mark prompted transcriptome-wide investigations of N6-methyladenosine. This modification was found to be widespread and enriched near stop codons, the 5′ and 3′ UTR and internal long exons of mRNA, as well as rRNA, tRNA, snoRNA and lncRNA (Meyer et al., 2012; Berulava et al., 2015). A canonical m^6^A motif was identified and consists of RRACH with *R* =G/A and *H* =A/C/U (Balacco and Soller, 2019; Dominissini et al., 2012). The methyl group is catalyzed from a donor substrate S-adenosylmethionine (SAM) to an adenosine residue of an RNA moiety along a specific sequence as stated above (Bokar et al., 1997). The m6A modification plays a role in several diverse RNA mechanisms, most notably RNA stability and translational efficiency (Meyer et al., 2015; Chen X.Y. et al., 2019). Other studies have implicated m6A in the control of mRNA dynamics including alternative splicing (although there is considerable debate regarding this Ke et al., 2017) and subcellular localization. Moreover, the role of m^6^A may be dictated by the subcellular localization of the m^6^A-tagged RNA. In the nucleus, m^6^A deposited on nascent pre-mRNA may influence alternative splicing (Dominissini et al., 2012), and microRNA biogenesis (Alarcon et al., 2015), while in the cytoplasm, it is thought to regulate RNA stability (Batista et al., 2014; Wang X. et al., 2014), translational efficiency and RNA decay (Wang et al., 2015).

## M^6^A Machinery

**FIGURE 1:**
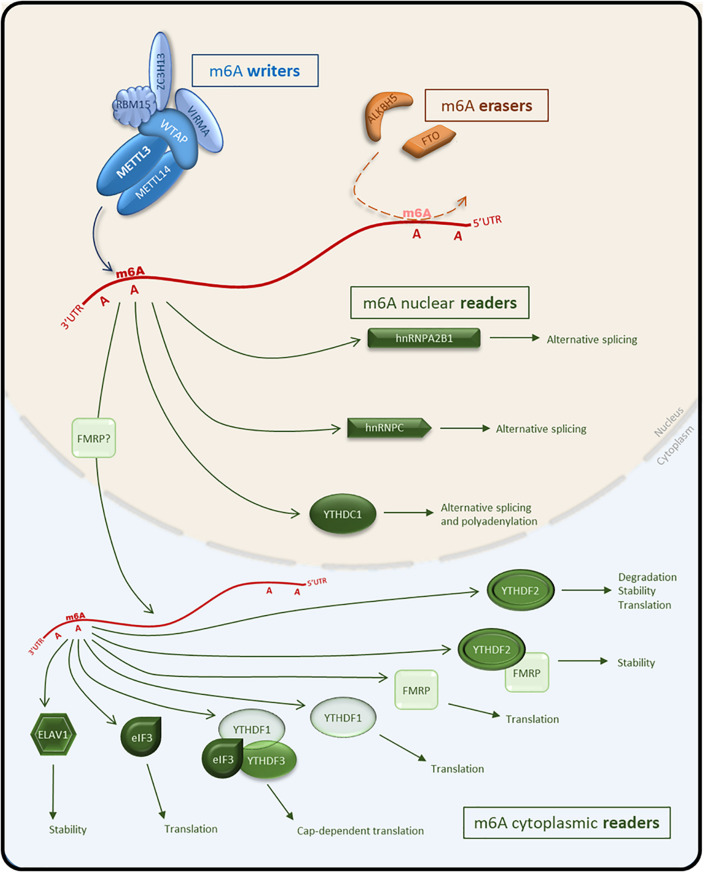
Schematic representation of the m^6^A pathway and effectors on mRNA. The MACOM complex composed of m^6^A writers (METTL3, METTL14, WTAP, VIRMA, ZC3H13, and RBM15) deposits m^6^A on target RNAs. M^6^A erasers (FTO and ALKBH5) remove the m^6^A mark. M^6^A nuclear readers (hnRNPC, hnRNPA2B1, and YTHDC1) facilitate alternative splicing or polyadenylation following recognition of m^6^A-tagged RNA. M^6^A-tagged RNA can be exported to the cytoplasm and bound by cytoplasmic readers (eIf3, ELAVL1, YTHDF1,2,3) to modulate stability, translational efficiency or the degradation of RNA. Blue, m^6^A writers; Orange, m^6^A erasers; Green, m^6^A readers.

M^6^A is a dynamic modification, catalyzed by a distinct enzymatic complex (writers), identified and processed by several “reader” proteins and potentially removed by “eraser” proteins. In the following sections, we summarize what is known about the proteins associated with the deposition, identification, and removal of m^6^A (Figure 1).

## M^6^A Writers

The methyltransferase complex which catalyzes m^6^A addition is composed of two distinct sub-complexes (Knuckles et al., 2018): the m^6^A-methyltransferase-like (METTL) complex (MAC) which is composed of METTL3 and METTL14 and the m^6^A-METTL associated complex (MACOM) which consists of RBM15, ZC3H13, WTAP, and VIRMA (Meyer and Jaffrey, 2014; Cao et al., 2016; Lence et al., 2019). Together MAC and MACOM function to catalyze the addition of methyl groups to adenosine.

### MAC Complex

The MAC-associated proteins comprise the catalytic components of the methyltransferase complex, which co-transcriptionally deposit m^6^A on target mRNAs (Liu et al., 2014). There are two essential components of the MAC complex, METTL3 and METTL14 which form a conserved heterodimeric core. This dimerization is essential for their methylation function and provides a synergistic effect on the catalytic activity of the complex (Liu et al., 2014; Balacco and Soller, 2019). Crystallographic studies of the MAC complex have shed light on the mechanisms of m^6^A deposition on target mRNA molecules (Sledz and Jinek, 2016). Additionally, transcriptome-wide profiling of m^6^A has identified a specific sequence motif known as a RRACH with *R* = G/A and *H* = A/C/U sequence within which m^6^A is usually confined. This RRACH sequence is highly conserved and restricts m^6^A to a selection of conserved transcripts (Dominissini et al., 2012). In some species, such as *C. elegans*, the dimer is replaced by a prologue, METTL4. Furthermore, METTL16, which is not a prologue, can also methylate mRNA but its mechanism of action remains to be elucidated (Balacco and Soller, 2019).

**METTL3** is the primary catalytic component of the MAC complex. Knockout of METTL3 has been reported to result in a significant reduction in global m^6^A levels (Batista et al., 2014; Wang Y. et al., 2014). METTL3 selectively targets mRNAs, depositing m^6^A on nascent RNA via its zinc-finger motifs (Batista et al., 2014; Sledz and Jinek, 2016). A mechanistic study in acute myeloid leukemia (AML) found that METTL3 is recruited by the transcription factor CHOP (CEBPζ) and methylates CHOP-regulated transcripts (Barbieri et al., 2017). METTL3 levels influence the expression of other m^6^A-associated writers. Indeed, both elevated and reduced levels of METTL3 lead to increased amounts of WTAP mRNA translation and promote the stabilization of the protein (Sorci et al., 2018). Furthermore, METTL3 can reportedly switch from writer to reader by translocating to the cytoplasm where it may regulate the translation of specific mRNAs by direct binding to RNA and recruitment of eIF3 (Lin et al., 2016).

**METTL14** has a functional role in structural stabilization and RNA substrate recognition (Sledz and Jinek, 2016). METTL14 enhances METTL3 methyltransferase activity by binding the mRNA and orientating the SAM methyl group for the reaction (Schwartz et al., 2014; Balacco and Soller, 2019). In the brain, METTL14 also plays an important role in maintaining neuronal populations via targeting transcripts of transcription factors of cell cycle to promote their decay (Yoon et al., 2017; Balacco and Soller, 2019). While it does not possess catalytic activity, the depletion of METTL14 causes profound reduction in m^6^A levels in embryonic stem cells, is embryonic lethal in mice and affects cortical development suggesting a critical role in development and highlighting its necessity for normal MAC activity (Wang Y. et al., 2014; Yoon et al., 2017).

### MACOM Complex

MACOM is an m^6^A-associated complex (Meyer and Jaffrey, 2014; Cao et al., 2016; Lence et al., 2019). It is thought that the proteins associated with the MACOM complex may regulate m^6^A deposition by integrating cellular signaling pathways and stimuli, dictating the repertoire of transcripts which undergo m^6^A-tagging (Livneh et al., 2020). The function of the MACOM is under intense investigation.

**WTAP** (Wilms’ tumor 1-associated protein) is a ubiquitously expressed protein and a regulatory subunit required for formation of a functional and stable MACOM complex (Ping et al., 2014). WTAP also modulates RNA processing, translation, and alternative splicing (Horiuchi et al., 2013). It was through the mass-spectrometric analysis of WTAP binding partners that several other methyltransferase enzymes were identified including METTL3-METTL14, RBM15 and VIRMA (Ping et al., 2014).

**VIRMA** (vir like m^6^A methyltransferase associated), also named KIAA1429, recruits the WTAP-METTL3-METTL14 complex via its binding to WTAP (Yue et al., 2018; Balacco and Soller, 2019). VIRMA may also interact with polyadenylation cleavage factors linking the machineries of m^6^A methylation and polyadenylation during mRNA processing (Yue et al., 2018).

**RBM15** (RNA-binding motif protein 15) interacts with METTL3 via WTAP. It is particularly associated with the regulation of m^6^A levels on lncRNAs (Patil et al., 2016; Balacco and Soller, 2019). RBM15 acts as an adaptor protein recruiting the m^6^A methylosome to U-rich regions.

**ZC3H13** (Zinc Finger CCCH-type containing 13), also named Flacc (Fl(2)d-associated complex component) (Wen et al., 2018; Balacco and Soller, 2019) may have a scaffolding function in the m^6^A methylosome. In Wen et al. (2018) found that ZC3H13 promotes pluripotency-associated gene expression and suppresses differentiation-associated genes in mESCs. Loss of ZC3H13 impairs WTAP-dependent 3′UTR m^6^A events. Moreover, Knuckles et al. have shown that ZC3H13 binds RBM15 and regulates the m^6^A pathway via the stabilization of the interaction between WTAP and RBM15 (Knuckles et al., 2018).

## M^6^A Erasers

Two proteins have been shown to possess m^6^A-demethylase activity. FTO (Fat mass and obesity-associated protein) and ALKBH5 (AlkB homologue 5 protein). These proteins are [(α-ketoglutarate (α-KG)-dependent dioxygenases which are inhibited by D2-hydroxyglutarate (D2-HG)] (Fedeles et al., 2015; Chen X.Y. et al., 2019).

FTO is an AlkB-like 2-oxoglutarate-dependent nucleic acid demethylase (Gerken et al., 2007), initially identified for its role in diabetes and obesity (Lindgren and McCarthy, 2008; Loos and Bouchard, 2008). Since then it has been shown that, in the nucleus, FTO catalyzes the removal of m^6^A and m^6^Am (*N*^6^, 2’-O-dimethyladenosine) marks from mRNA although importantly shows a 100-fold greater affinity for m^6^Am than m^6^A (Mauer et al., 2017; Mauer and Jaffrey, 2018; Balacco and Soller, 2019). The m^6^Am mark is a terminal modification distinct from the internal m^6^A mark and follows the m^7^G (*N*^7^-methylguanosine) cap at the *N*^6^ position (Wei et al., 1975), where A or m^6^A are not found (Wei et al., 1976). Some studies have identified FTO as an important regulator of m^6^A-tagged transcripts, important for mRNA alternative splicing and gene expression, contributing to the regulation of adipogenesis (Zhao et al., 2014; Bartosovic et al., 2017; Chen X.Y. et al., 2019). However, some recent emerging data does not support the role of FTO as an m^6^A eraser and suggests that FTO under normal physiological conditions does not remove m^6^A and solely functions on m^6^Am (Mauer et al., 2017). Indeed, FTO is present almost exclusively in the nucleus, which suggests it is unlikely to dynamically regulate cytosolic mRNAs. Furthermore, studies by the Darnell group have shown that the levels of m^6^A are perhaps stable once deposited. Together, this calls into question the extent of m^6^A demethylation *in vivo*, at least under normal physiological conditions (Mauer et al., 2017; Mauer and Jaffrey, 2018). M^6^A removal by FTO or others may therefore be context and cell-type specific and/or developmentally regulated and this warrants further investigation.

**ALKBH5** is an α-ketoglutarate dependent oxidase and nuclear demethylase only found in vertebrates which can bind single stranded nucleic acids (Zheng et al., 2013; Balacco and Soller, 2019). In Ensfelder et al. (2018) identified ALKBH5 as a ribosomal RNA m^6^A eraser. It has since been shown that ALKBH5 is also required for correct splicing and production of transcripts in spermatocytes via selective removal of m^6^A (Tang et al., 2018). Moreover, ALKBH5 is involved in glioblastoma to repress tumorigenesis (Zhang et al., 2017), in spermatogenesis and in male fertility (Zheng et al., 2013). Again, the relevance of ALKBH5 as an m^6^A eraser in somatic cells remains unclear. The study by Darnell did note that there was loss of m^6^A on a minority of transcripts suggesting a small amount of selective demethylation may occur under normal physiological conditions (Ke et al., 2017).

## M^6^A Readers

M^6^A readers elicit different actions upon recognition and binding of the methylated RNA transcripts. Several reader proteins have now been identified.

**HNRNPC & HNRNPA2B1** (Heterogeneous Nuclear Ribonucleoprotein C & A2B1) are both nuclear-confined m^6^A readers and are particularly involved in pre-mRNA processing and in maturation of pri-miRNA to pre-miRNA (Cao et al., 2016). HNRNPA2B1 promotes processing of METTL3-dependent microRNAs and plays a role in the regulation of the alternative splicing of exons (Alarcon et al., 2015).

**YTH domain containing proteins** (YTHDC1 and YTHDC2) and **YTH domain-containing family proteins** (YTHDF1, YTHDF2 and YTHDF3), a highly conserved family of proteins, have been shown to detect and bind m^6^A (Berlivet et al., 2019). The DC proteins are usually confined to the nucleus (Balacco and Soller, 2019) while the DF proteins are found primarily in the cytoplasm. The structure of m^6^A-bound by YTH-domain containing proteins was elucidated several years ago. The YTH domain is critical for this binding and is involved in the development of a tryptophan cage around the m^6^A mark (Theler et al., 2014; Xu et al., 2014). Recent iCLIP data from the Jaffrey lab, identified the binding sites for all YTH proteins. They found that DC1 binds m^6^A sites in mRNA and nuclear non-coding RNAs, while all three DF proteins bind most m^6^A sites (Patil et al., 2018). It has been found that DC1 contributes to extensive alternative polyadenylation and 3′UTR length alteration (Chen X.Y. et al., 2019) while DC2 is primarily localized in the testes where it has been found to have weak m^6^A affinity (Wojtas et al., 2017). In the mouse hippocampus, upon binding, YTHDF1 promotes the translation of m^6^A-tagged transcripts via interaction with a translation initiation factor (Wang et al., 2015) and the help of YTHDF3 (Shi et al., 2017) and of eIF1 (Chen X.Y. et al., 2019) to facilitate learning and memory (Shi et al., 2018). YTHDF2 recognition of m^6^A sites, contrarily, was reported to regulate the degradation of specific mRNAs via the recruitment of CCR4-NOT (Alarcon et al., 2015; Cao et al., 2016; Du et al., 2016; Chen X.Y. et al., 2019). This protein may also play a role in mRNA stability and translation (Wang X. et al., 2014; Chen X.Y. et al., 2019), targeting stop codon regions, 3′UTR and coding regions (Wang X. et al., 2014). Recent studies however, including iCLIP-sequencing datasets suggest that all three DF proteins do not selectively bind to m^6^A-tagged transcripts and all likely lead to degradation of bound transcripts. It is possible that context dependence is critical toward understanding the function of m^6^A-recognition by YTH-domain containing proteins.

**METTL16**, usually a writer enzyme, may also function as a reader when SAM levels are low, in order to stimulate the SAM synthetase MAT2A mRNA translation to increase the level of SAM. In contrast, when SAM levels are high, METTL16 methylates MAT2A mRNA, which promotes nuclear mRNA decay (Pendleton et al., 2017; Balacco and Soller, 2019).

**elF3** (eukaryotic initiation Factor 3) is a component of the 43s translation pre-initiation complex. It has been found that this protein is an m^6^A reader, which promotes translation via two mechanisms. First, eIF3 can directly bind the 5′UTR-localised m^6^A to allow the recruitment of the 43s complex (Meyer et al., 2015; Cao et al., 2016). The second method is via its interaction with YTHDF1 which facilitates cap-dependent translation (Wang et al., 2015).

**FMRP** (Fragile X mental retardation protein) is an RNA-binding protein, loss of which is implicated in the neurodevelopmental condition Fragile X syndrome (FXS). It has been shown to be important for the stability of m^6^A-tagged mRNA (Zhang et al., 2018) and for their nuclear export (Hsu et al., 2019). FMRP has been found to interact with WTAP and recently suggested to bind m^6^A sites by competing with YTHDF2 to regulate specific mRNA turnover (Horiuchi et al., 2013; Balacco and Soller, 2019). Indeed, another study found that FMRP modulates the stability of its target through YTHDF2 (Zhang et al., 2018). FMRP was recently shown to directly bind to m^6^A-marked mRNA (Westmark et al., 2020) and negatively regulate their translation (Edupuganti et al., 2017). How m^6^A processing in FXS is affected has yet to be analyzed.

**ELAVL1** (Drosophila homologue-like 1), also known as human antigen R (HuR), and IGF2BPs (Insulin-like growth factor 2 mRNA-binding proteins) has been identified as an m^6^A reader (Balacco and Soller, 2019). Several studies show that ELAVL1 increases the stability of transcripts via m^6^A-mRNA-binding. Indeed, in prostatic carcinoma cells, ELAVL1 plays a role in stabilizing integrin β1 mRNA (Li et al., 2020). It may also promote SOX2 mRNA stabilization to stimulate the maintenance of glioma stem-like cells (Visvanathan et al., 2018) as well as DRG1 in osteosarcoma human tissue (Ling et al., 2020).

## M^6^A in the Brain

The m^6^A mark influences the behavior of RNA in several biological pathways and subsequently biological processes like cell differentiation and proliferation, development, sex determination and circadian rhythms (Chen et al., 2017). Here, we review the most important and studied cerebral processes impacted by m^6^A methylation such as learning and memory as well as neurogenesis, neurodevelopment, the stress response, myelination, and axon plasticity (Table 1).

**TABLE 1:** m^6^A machinery proteins associated in Neurological functions.

**Neurological function**	**Related proteins**	**Explication**	**References**
Learning and memory	METTL3	Increased METTL3 after cued fear conditioning in mPFC. Knockdown of METTL3 increases the time of memory consolidation process.	Widagdo et al., 2016; Zhang et al., 2018
	METTL14	Knockdown of METTL14 in striatal neurons decreases m6A levels and impairs learning in mice.	Koranda et al., 2018
	FTO	Decreased FTO following cued fear conditioning in CA1 of hippocampus. Knockdown of FTO increased m^6^A levels and enhanced memory retention.	Walters et al., 2017
	YTHDF1	YTHDF1 promotes translation of memory association transcripts. Knockdown of YTHDF1 impairs memory formation.	Shi et al., 2018
Neurogenesis, neurodevelopment and plasticity	METTL14	Knockdown of METTL14 extends cortical neurogenesis in post natal stage. Ablation of METTL14 decreases the maturation of oligodendrocyte and causes abnormal myelination.	Donega et al., 2018; Xu H. et al., 2020
	FTO	FTO depletion reduces proliferation and differentiation of aNSCs. FTO regulates m^6^A in axonal GAP-43 mRNA.	Li et al., 2017; Yu et al., 2018
	YTHDF2	Loss of YTHDF2 reduces basal progenitor cells and impairs neuronal differentiation.	Li et al., 2018
	YTHDF1	YTHDF1 allows synthesis of proteins for axonal regeneration by enhancing mRNA translation.	Weng et al., 2018
Stress response regulation	METTL3	Knockout of METTL3 disrupts stress behavior.	Engel et al., 2018
	FTO	Knockout of FTO disrupts stress behavior.	Engel et al., 2018
	YTHDF2	After heat shock stress, YTHDF2 limits m^6^Ademethylation by FTO.	Zhou et al., 2015

*mPFC, medial PreFrontal Cortex; CA1, Cornus Ammonis1; aNSCs, adult Neural Stem Cells. Blue, m^6^A writers; Orange, m^6^A erasers; Green, m^6^A readers.*

### Learning and Memory

In Widagdo et al. (2016) performed the first exploration of m^6^A in synaptic plasticity and memory processes. To assess whether m^6^A was regulated by experience, they used a cued-fear conditioning model in mice and found extensive experience-induced m^6^A changes using meRIP-Seq. Additionally, they found an increase in m^6^A marks on mRNA in the medial prefrontal cortex (mPFC) following the cued-fear conditioning paradigm as well as increased levels of METTL3 suggesting a critical role for m^6^A in behavioral adaptation. A subsequent study also using a fear-conditioning paradigm, found decreased levels of FTO in dorsal CA1 hippocampal neurons following fear-conditioning (Walters et al., 2017). Subsequent knock-down of FTO, in mPFC or in the CA1 increased m^6^A levels (Walters et al., 2017) and enhanced memory retention suggesting m^6^A plays a critical role in memory formation and consolidation (Widagdo et al., 2016; Walters et al., 2017). Zhang et al. found that conditional postnatal depletion of *METTL3* in hippocampus of mice prolonged the process of memory consolidation but did not alter short-term plasticity. Furthermore, restitution of *METTL3* improved learning while the overexpression of *METTL3* with a mutated methyltransferase domain had no effect (Zhang et al., 2018). Together this suggests that METTL3 participates in the enhancement of long-term memory consolidation via its m^6^A methyltransferase function (Zhang et al., 2018). Moreover, another study showed that m^6^A methylation promotes learning and memory through YTHDF1, which boosts translation of memory-associated transcripts. Indeed, the depletion of YTHDF1 impairs long-term potentiation of hippocampal synapses leading to impairment of memory formation (Shi et al., 2018). The METTL3/YTHDF pathway is also required for memory formation in *Drosophila*. *METTL3* knockdown in the mushroom body, impaired memory as assessed using an aversive conditioning paradigm to assess short-term memory. They further identified that YTHDF hemizygotes exhibited age-related memory impairments similar to *METTL3* knockdown flies. Furthermore, METTL14 deletion in striatal neurons induces a decrease of m^6^A methylation and impairs learning in mice (Koranda et al., 2018).

Local supply of mRNA, microRNAs and translational machinery facilitate rapid synaptic alterations required for learning and memory. Recently, Merkurjev et al. (2018) demonstrated synaptic enrichment of several m^6^A-associated enzymes including METTL14, and YTHDF1-3 as well as m^6^A-tagged polyA RNA. The authors isolated and profiled synaptosomal m^6^A-tagged RNA using a low-input meRIP-Seq approach to reveal that the most significant biological processes represented by synaptic m^6^A-tagged RNAs were those critical for neuronal integrity and function, suggesting m^6^A-tagged synaptic RNAs critically modulate neuronal function. Furthermore, disruption of m^6^A-processing via knockdown of reader proteins YTHDF1 or YTHDF3 resulted in significant morphological disruptions including increased spine neck length and decreased spine head width, reduced PSD-95 clustering, and reduced surface expression of AMPA receptors (Merkurjev et al., 2018).

Other studies have also revealed a potential role for m^6^A-tagged RNAs in synaptic function. Indeed, several experimental paradigms have been shown to alter methylation of synapse associated RNAs including cocaine exposure, depression, and deep brain stimulation (Madugalle et al., 2020; Song et al., 2020; Xue et al., 2021).

Although further studies are required, these initial data suggest that m^6^A may represent a critical mechanism of mRNA translational priming and enable rapid sorting of mRNAs required for synaptic function.

### Neurogenesis/Neuronal Development

Neurogenesis, the process by which new neurons are formed, is critical for correct neurodevelopment, function and repair. Furthermore, aberrant neurogenesis is associated with several neurological diseases including epilepsy (Danzer, 2008; Livneh et al., 2020). Selective deletion of M*ETTL14* in embryonic mouse brains expands cortical neurogenesis into the postnatal stage, extends the cell cycle progression in neuronal progenitor cells and prolongs maintenance of radial glia cells (Yoon et al., 2017). This suggests m^6^A has an important role during brain development. During pre-natal cortical development m^6^A-tagging of developmental transcription factors Pax6, Sox2 and Neurogenin2 is enriched and promotes turnover allowing rapid and complex temporal regulation of gene expression critical for development (Donega et al., 2018). M^6^A levels and deposition are dynamically regulated during post-natal cerebellum development which correlates with spatiotemporal regulation of m^6^A-associated enzymes including METTL3, 14, and FTO (Ma et al., 2018). Importantly, transcripts essential for development in post-natal cerebellum were continuously and consistently methylated whereas time-specific processes were temporarily methylated inducing proper cerebellar development as disruption of either METTL3 or FTO caused developmental deficits. Furthermore, it has been shown that FTO deficiency reduces the proliferation and differentiation of adult neural stem cells (aNSCs) leading to a smaller brain in mice (Li et al., 2017). While m^6^A writers have emerged as important mediators of neuronal development, the role of m^6^A readers is less clear. Recently, Li and colleagues demonstrated that loss of YTHDF2 is embryonic lethal and found that neuronal development dysfunction resulting from YTHDF2 loss prevented viability. Analysis of embryonic brains from E12.5 to E14.5 mice identified that YTHDF2 loss resulted in reduced cortical layering and reduced cortical development. Further analysis revealed reduced basal progenitor cells and impaired neural differentiation (Li et al., 2018).

M^6^A methylation has been found to be essential for the maturation of oligodendrocytes and for central nervous system myelination. In mice, in the early postnatal stage, METTL14 ablation leads to a decrease of oligodendrocyte maturation and causes abnormal myelination. *In vitro*, METTL14 depletion induced a diminution of oligodendrocyte differentiation and a prolongation of the cell cycle progression. This indicates a role for m^6^A methylation in neuronal development, transmission and plasticity (Xu H. et al., 2020).

In addition to a role in neuronal development, m^6^A may also play a crucial role in neuronal repair and in axonal regeneration. In peripheral nerves, it was shown that m^6^A allows the translation of retrograde signaling molecule mRNAs which enables rapid axonal regeneration. Following axonal injury, m^6^A levels are increased, which promotes the synthesis of proteins involved in axon regeneration and recovery, through YTHDF1, to enhance mRNA translation (Weng et al., 2018). Moreover, FTO is enriched in axons leading to a regulation of m^6^A in axonal *GAP-43* mRNA, which is required for axon elongation (Yu et al., 2018). Further research is now required to determine whether the m^6^A pathway may be exploited to improve nerve regeneration following physical injury.

### Stress Response Regulation

Physical and emotional stressors are known to influence transcriptional and translational output and significantly impact cognitive function long-term (Short and Baram, 2019). Recently, studies have also shown that m^6^A-mediated gene regulation is significantly affected by stress. The dynamic nature of the stress response was recently demonstrated by Engel and colleagues, using a restraint stressor, a stressful stimulus for rodents. This elicited large-scale changes in the RNA methylome in a temporospatial manner (Engel et al., 2018). Restraint stress led to an increase in global methylation levels in amygdala and a decrease in prefrontal cortex. Mice deficient in METTL3 and FTO from excitatory hippocampal cells displayed lower resilience to stressful stimuli suggesting m^6^A-mediated mRNA regulation may improve adaptation to stress. These results highlight a role of m^6^A in stress response.

### Heat Shock Stress Response

Zhou et al. found that m^6^A levels change in response to heat shock stress in both mouse embryonic fibroblasts (MEFs) and HeLa cells. They detected more m^6^A deposition in the 5′UTR of mRNAs, which promotes cap-independent translational initiation, allowing selective translation of heat-shock response proteins. YTHDF2 plays a key role in maintaining this 5′UTR methylation by preventing FTO-mediated demethylation (Zhou et al., 2015).

## M^6^A in Brain Disorders

As described above, m^6^A RNA methylation is involved in many essential cerebral processes, so, unsurprisingly, this process is found to be altered in many brain disorders. Here we describe neurological disorders in which m^6^A, or m^6^A-associated proteins are affected such as Alzheimer’s disease, Parkinson’s disease, glioblastoma, schizophrenia, depression, transient focal ischemia, and traumatic brain injury (Table 2).

**TABLE 2:** m^6^A machinery proteins in neurological diseases.

**Neurological diseases**	**Related proteins**	**Explication**	**References**
Alzheimer’s disease	METTL3	Increased METTL3 levels in AD mice. Aberrant METTL3 expression in human post-mortem brain of AD patient.	Han et al., 2020; Huang et al., 2020
	FTO	Genetic variation in *FTO* associated with AD risk.	Reitz et al., 2012
	Unknown	Variation of m^6^A levels in cortex and hippocampi of AD mice.	Han et al., 2020
Glioblastoma	METTL3	METTL3, via m^6^A methylation, regulates the growth and renewal of cancer cells.	Cui et al., 2017
	ALKBH5	Overexpression of ALBKH5 in patient-derived glioblastoma stem cells.	Kowalski-Chauvel et al., 2020; Yarmishyn et al., 2020
	YTHDF1	Overexpression of YTHDF1 to promote proliferation of glioblastoma cells.	Dixit et al., 2020
	YTHDF2	Overexpression of YTHDF2 to regulate glucose metabolism in glioblastoma stem cells.	Wang L.C. et al., 2020
	HNRNPC	Overexpression of HNRNPC to promote glioma progression.	
Neuroblastoma	METTL14	SNPs in METTL14 gene associated with predisposition to neuroblastoma. High expression of METTL14 associated with low survival of patient.	Wang Z. et al., 2020; Zhuo et al., 2020
	WTAP	Low expression of WTAP associated with low survival of patient.	Wang Z. et al., 2020
	YTHDF1	High expression of YTHDF1 associated with low survival of patient	Wang Z. et al., 2020
Parkinson’s disease	FTO	Increased FTO in midbrain of PD rat model.	Chen X. et al., 2019
	ALKBH5	SNPs identified in ALKBH5 gene in PD patients.	Qiu et al., 2020
Transient focal ischemia	METTL3	METTL3 promotes stress granule formation (neuroprotective).	Si et al., 2020
	FTO	Decreased FTO after stoke.	Chokkalla et al., 2019
	YTHDC1	Increase of YTHDC1 following ischemia in rat supports neuronal survival.	Zhang et al., 2020
	YTHDF1	Decreased YTHDFL following ischemia in rat limits inflammation.	Zhang et al., 2020
Traumatic brain injury	METTL3	Decreased METTL3 in TBI rat model.	Yu et al., 2020
	METTL14	Decreased METTL14 levels in TBI rat model.	Yu et al., 2020
	FTO	Decreased FTO levels in TBI rat model.	Yu et al., 2020

*AD, Alzheimer’s disease; PD, Parkinson’s disease; TBI, Traumatic Brain Injury. Blue, m^6^A writers; Orange, m^6^A erasers; Green, m^6^A readers.*

### Neurodegenerative Diseases

Several studies in recent years have linked m^6^A involvement with neurodegenerative processes. Keller found that FTO is associated with dementia-like Alzheimer’s disease (AD) risk, suggesting that FTO may interact with the AD-risk factor gene APOE (Keller et al., 2011). Follow up studies have found that m^6^A is indeed dysregulated in AD. M^6^A levels were found to be disrupted in the cortex of APP/PS1 AD mice (Han et al., 2020), specifically they found gross changes in the RNA methylome with enhanced m^6^A levels on 659 gene transcripts and depleted m^6^A on 991 gene transcripts. Additionally, they found increased METTL3 expression in AD mice while FTO expression was decreased (Han et al., 2020). Aberrant METTL3 expression has been shown in human post-mortem brain of AD patients compared to non-AD autopsy tissue, further confirming mouse model data and strengthening the potential involvement of m^6^A-associated gene expression dysregulation in AD (Huang et al., 2020). Further investigations are needed however to determine whether aberrant m^6^A is a causal factor in AD development and progression or a consequence of altered physiological processes in the diseased brain.

M^6^A levels were reduced in the striatum of rats with 6-OHDA-induced Parkinson’s disease (PD) potentially due to an upregulation of ALKBH5. This reduction in m^6^A may promote the expression of N-methyl-D-aspartate receptor 1 (NMDA) and a subsequent elevation of oxidative stress and Ca^2+^ influx. This molecular cascade may then promote excitotoxic cell death of dopaminergic neurons (Chen X. et al., 2019). Since then, five Parkinson’s Disease-associated m^6^A-SNPs were identified in PD patients which perturbed this pathway, three of these SNPs were identified in the *ALKBH5* gene (Qiu et al., 2020).

Together these data highlight the complex interplay amongst m^6^A associated proteins and suggest that further research investigating how m^6^A may actively contribute to perturbed gene expression regulation in neurodegenerative diseases is required.

### CNS Tumors

M^6^A has been found to regulate glioblastoma stem cell tumorigenesis by controlling the expression of cancer-associated genes and processes. As in several other forms of cancer, m^6^A is thought to regulate the growth and self-renewal of cells via the methyltransferase catalytic activity of METTL3 (Cui et al., 2017). M^6^A readers may also play a critical role in glioblastoma tumorigenesis, indeed HNRNPC, YTHDF1 and YTHDF2 are all expressed at elevated levels in glioblastoma (Wang L.C. et al., 2020). YTHDF2 has been found to regulate glucose metabolism via the stabilization of the proto-oncogene *MYC* in glioblastoma stem cells (Dixit et al., 2020). Elevated YTHDF1 was associated with poor prognosis as it is thought to promote the proliferation and migration of glioblastoma cells (Yarmishyn et al., 2020). HNRNPC contributes to glioma progression although the mechanisms underlying this association are not yet known (Wang L.C. et al., 2020). The m^6^A-demethylase *ALKBH5* has been shown to be overexpressed in patient-derived glioblastoma stem cells and may influence radio-resistance via regulation of DNA damage response genes including Chk1 (Kowalski-Chauvel et al., 2020).

M^6^A RNA modification has also been associated with other brain cancers such as neuroblastoma. Zhuo et al. identified several SNPs in the gene encoding *METTL14*, which may be associated with a predisposition to neuroblastoma development in a Chinese population (Zhuo et al., 2020). Moreover, m^6^A and associated proteins may also represent potential biomarkers of various brain cancers including neuroblastoma. High expression of *METTL14* was correlated with low survival of neuroblastoma patients along with reduced expression of *WTAP* or a high expression of *YTHDF1* (Wang Z. et al., 2020).

### Brain Injury

Ischemic stroke resulting from blood vessel occlusion deprives neurons and brain cells of oxygen and nutrients and causes pronounced neuronal damage in affected tissues. Recently, Chokkalla et al. found using immunoprecipitation of m^6^A followed by microarray analysis that following ischemic stroke in mice, there is an increase in m^6^A levels in the brain and levels are perturbed particularly on inflammation, apoptosis and transcription-associated transcripts suggesting involvement of m^6^A in the regulation of the molecular milieu initiated by stroke. Elevated levels of m^6^A following stroke may be explained by decreased expression of FTO levels (Chokkalla et al., 2019; Xu K. et al., 2020).

Using the middle cerebral artery occlusion (MCAO) model of ischemic stroke in rats, Si and colleagues found that METTL3-mediated methylation promoted maturation of miR-355 which then promoted stress granule formation which was neuroprotective (Si et al., 2020). A role for m^6^A readers as regulators of the brain response to stroke has also been proposed. YTHDC1 levels increased following ischemia in rats while YTHDF1, 2, and 3 levels were repressed (Zhang et al., 2020). Elevated YTHDC1 levels may support post-ischemic neuronal survival via the modulation of the Akt/PTEN pathway (Zhang et al., 2020). YTHDF1 reduction may be a protective or adaptive mechanism by limiting post-stroke inflammation (Zheng et al., 2020).

Following traumatic brain injury (TBI) in rats, *METTL3, METTL14* and *FTO* expression are all downregulated in the cortex. m^6^A methylation levels are also significantly changed with upregulated m^6^A-tagging of some mRNA (370 transcripts) and a downregulation of others (552 transcripts) as detected using meRIP-Seq (Wang et al., 2019; Yu et al., 2020). The exact role of m^6^A following TBI has yet to be fully elucidated but initial reports suggest these investigations are warranted.

## Enabling Technologies

As discussed, m^6^A was identified as a prevalent RNA modification as far back as the 1970s yet was largely ignored until recently due to a lack of resources available to profile and probe its function (Munns et al., 1977; Wei and Moss, 1977; Burke and Joh, 1982). The development of an m^6^A specific antibody coupled with the growing use of next generation sequencing in the last decade led to the development of methylated RNA immunoprecipitation sequencing (meRIP-Seq/m^6^A-Seq) approaches (Dominissini et al., 2013). This technology is an adaptation of a standard RIP-Seq approach whereby RNA is isolated from a sample and sheared to a specific length. RNA is ribosome depleted and/or polyA-selected and then subjected to immunoprecipitation to isolate m^6^A-tagged mRNAs. The resulting isolated m^6^A-tagged RNA is then used to prepare RNA-Seq libraries and subjected to next generation sequencing and whole transcriptome analysis. The development of this technique enabled widespread analysis of this modification in various tissues, organisms and disease settings and highlighted the extent of the epitranscriptomic modification as well as identify how it may contribute to disease. Certain limitations existed however including high RNA input requirements (300 (μg total RNA) and it also suffers from limited resolution although bioinformatic predictions do help to identify exact sites of m6A. These limitations have been overcome to some degree and meRIP-seq can now be performed with as little as 3 (μg starting material (Zeng et al., 2018). Furthermore, single base resolution of m6A can now be mapped using crosslinking and immunoprecipitation (miCLIP) approaches (Linder et al., 2015).

M^6^A can now be mapped in native full length RNA moieties without the need for reverse transcription, shearing or immunoprecipitation steps using nanopore long read sequencing technologies (Liu et al., 2019; McIntyre et al., 2019; Jenjaroenpun et al., 2021). Several robust bioinformatic pipelines to identify methylated bases from long sequencing reads have now also been established.

## Future Directions

The complex functions of the mammalian brain are dependent upon precise control of gene expression and regulation in a temporospatial fashion. M^6^A represents an additional layer of gene regulation, which has now been shown to critically regulate neuronal development and function. Technically difficult to study, tools are now available to analyze m^6^A RNA methylation at a global level and at single base resolution and there is huge scientific interest in this modification at present (Dominissini et al., 2012; Linder and Jaffrey, 2019). The next hurdle lies in developing and adapting current applications to profile m^6^A profiles in discrete cell types and populations/brain regions to understand how it contributes to neurodevelopment at the single cell level as well as specific brain functions such as learning and memory. Further challenges lie in understanding the precise function of m^6^A-eraser proteins in a context-dependent manner as well as probing the regulatory mechanisms governing m^6^A-associated protein expression and function.

Understanding the role of m^6^A in physiological homeostasis and disease is critical as components of these pathways may represent therapeutic targets for the treatment of neurological disease including many underserved conditions like TBI and stroke. The development of specific pharmacological inhibitors and antisense oligonucleotide approaches will also enable more precise interrogation of transient manipulations of m^6^A-associated proteins and therapeutic viability.

## Author Contributions

JM, DH, and GB researched and wrote the article. All authors contributed to the article and approved the submitted version.

## Conflict of Interest

The authors declare that the research was conducted in the absence of any commercial or financial relationships that could be construed as a potential conflict of interest.
